# Influence of vasopressor agent in pediatric septic shock mortality

**DOI:** 10.1186/cc10164

**Published:** 2011-06-22

**Authors:** CMF Mangia, LPS Jose, FL Monteiro, AT Fernandes, P Biasi, F Menezes, EL Lima, C Oliveira, F Bueno, P Paiva, MC Andrade

**Affiliations:** 1 Universidade Federal de São Paulo, São Paulo - SP, Brazil

## Objective

To clarify the impact of the choice of vasopressor support on mortality in pediatric septic shock (SS).

## Methods

A retrospective study based on the institutional database analyzing 1,050 patients admitted from October 1999 to January 2005. We studied children with SS after the neonatal period admitted to the pediatric intensive care (PICU) and we assessed the vasopressor support in the first 24 hours, PICU and hospital (HSP) length of stay (LOS), number of vasoactive drugs used, association between drugs and HSP mortality.

## Results

There were 101 consecutive patients with SS, mean age 41 months (95% CI = 30 to 52 months); mean of PICU LOS 16.73 days (95% CI = 11.18 to 22.28) and hospital LOS 55.46 days (95% CI = 43.16 to 67.75). PICU mortality was 32% and HSP mortality after PICU discharge was 10.8%. Of these, 33% patients received dobutamine and 26% patients dopamine as the only vasoactive drug. Dopamine plus dobutamine was used in 17.8%; dobutamine plus norepinephrine in 18% and dopamine plus norephinephrine in 3.9%. The HSP mortality associated with dobutamine was 29.4%; dopamine 53.8%; dopamine plus dobutamine 50%; dopamine plus norepinephrine 25%. The dopamine and dopamine plus dobutamine groups had higher hospital mortality (66% vs. 34%). Dopamine was associated with hypertensive state (odds ratio, 0.433; 95% CI = 0.192 to 0.976; *P *= 0.047), hypoxemia (odds ratio, 0.190; 95% CI = 0.040 to 0.909) and mechanical ventilation utilization (odds ratio, 2.625; 95% CI = 1.085 to 6.327; *P *= 0.035).

## Conclusion

Adrenergic support for pediatric patients with SS remains controversial. A prospective randomized controlled trial will be important to determine which subgroups of SS patients will benefit most with each drug.

